# Minimisation of dialysis risk in hospital patients with chronic kidney disease (MinDial): study protocol for a multicentre, stepped-wedge, cluster-randomised controlled trial

**DOI:** 10.1186/s13063-024-08182-x

**Published:** 2024-06-07

**Authors:** D. Stelzer, H. Binder, M. Glattacker, E. Graf, M. Hahn, M. Hollenbeck, K. Kaier, B. Kowall, N. Kuklik, G. Metzner, N. Mueller, L. Seiler, S. Stolpe, C. Blume

**Affiliations:** 1 Institute of Medical Biometry and Statistics, Faculty of Medicine and Medical Center, University of Freiburg, Stefan-Meier-Str. 26, Freiburg, 79104 Germany; 2 Section of Health Care Research and Rehabilitation Research, Institute of Medical Biometry and Statistics, Faculty of Medicine and Medical Center, University of Freiburg, Hugstetter Straße 49, Freiburg, 79106 Germany; 3 Institute of Technical Chemistry, Leibniz University Hannover, Callinstraße 5, Hannover, 30167 Germany; 4 KfH Foundation for Preventive Medicine, Martin-Behaim-Straße 20, Neu-Isenburg, 63263 Germany; 5 Knappschaftskrankenhaus Bottrop GmbH, Academic Teaching Hospital of the University of Duisburg-Essen, Osterfelder Straße 157, Bottrop, 46242 Germany; 6 Knappschafts-Kliniken Service GmbH (KKSG), In der Schornau 23-25, Bochum, 44892 Germany; 7 Institute for Medical Informatics, Biometry and Epidemiology, University Hospital Essen, Hufelandstraße 55, Essen, 45147 Germany; 8 Centre for Clinical Trials Essen, University Hospital Essen, Hufelandstraße 55, Essen, 45122 Germany

**Keywords:** Chronic kidney disease (CKD), Kidney failure risk equation (KFRE), End-stage renal disease (ESRD), Estimated glomerular filtration rate (eGFR), Stepped-wedge design, Cluster-randomised trial (CRT)

## Abstract

**Background:**

Early identification of patients with chronic kidney disease (CKD) and advancing kidney insufficiency, followed by specialist care, can decelerate the progression of the disease. However, awareness of the importance and possible consequences of kidney insufficiency is low among doctors and patients. Since kidney insufficiency can be asymptomatic even in higher stages, it is often not even known to those belonging to risk groups. This study aims to clarify whether, for hospitalised patients with advanced chronic kidney disease, a risk-based appointment with a nephrology specialist reduces disease progression.

**Methods:**

The target population of the study is hospitalised CKD patients with an increased risk of end-stage renal disease (ESRD), more specifically with an ESRD risk of at least 9% in the next 5 years. This risk is estimated by the internationally validated Kidney Failure Risk Equation (KFRE). The intervention consists of a specific appointment with a nephrology specialist after the hospital stay, while control patients are discharged from the hospital as usual. Eight medical centres include participants according to a stepped-wedge design, with randomised sequential centre-wise crossover from recruiting patients into the control group to recruitment to the intervention. The estimated glomerular filtration rate (eGFR) is measured for each patient during the hospital stay and after 12 months within the regular care by the general practitioner. The difference in the change of the eGFR over this period is compared between the intervention and control groups and considered the primary endpoint.

**Discussion:**

This study is designed to evaluate the effect of risk-based appointments with nephrology specialists for hospitalised CKD patients with an increased risk of end-stage renal disease. If the intervention is proven to be beneficial, it may be implemented in routine care. Limitations will be examined and discussed. The evaluation will include further endpoints such as non-guideline-compliant medication, economic considerations and interviews with contributing physicians to assess the acceptance and feasibility of the intervention.

**Trial registration:**

German Clinical Trials Register DRKS00029691. Registered on 12 September 2022.

## Administrative information

Note: the numbers in curly brackets in this protocol refer to SPIRIT checklist item numbers. The order of the items has been modified to group similar items (see http://www.equator-network.org/reporting-guidelines/spirit-2013-statement-defining-standard-protocol-items-for-clinical-trials/).

**Table:** 

Title {1}	Minimisation of dialysis risk in hospital patients with chronic kidney disease (MinDial): study protocol for a multicentre, stepped-wedge, cluster-randomised controlled trial
Trial registration {2a and 2b}.	DRKS00029691 [German Clinical Trials Register; registered on 12 September 2022; https://drks.de/search/en/trial/DRKS00029691]
Protocol version {3}	Version 3, November 2023
Funding {4}	This research is funded by the Innovation Fund of the Federal Joint Committee (G-BA) which is a decision-making body of the joint self-administration of physicians, dentists, hospitals and health insurance funds in Germany, grant number 01NVF20028.
Author details {5a}	• D. Stelzer: Institute of Medical Biometry and Statistics, Faculty of Medicine and Medical Center, University of Freiburg, Germany • H. Binder: Institute of Medical Biometry and Statistics, Faculty of Medicine and Medical Center, University of Freiburg, Germany • M. Glattacker: Section of Health Care Research and Rehabilitation Research, Institute of Medical Biometry and Statistics, Faculty of Medicine and Medical Center, University of Freiburg, Germany • E. Graf: Institute of Medical Biometry and Statistics, Faculty of Medicine and Medical Center, University of Freiburg, Germany • M. Hahn: Knappschafts-Kliniken Service GmbH (KKSG), Germany • M. Hollenbeck: Knappschaftskrankenhaus Bottrop GmbH, Academic teaching hospital of the University of Duisburg-Essen, Germany • K. Kaier: Institute of Medical Biometry and Statistics, Faculty of Medicine and Medical Center, University of Freiburg, Germany • B. Kowall: Institute for Medical Informatics, Biometry and Epidemiology, University Hospital Essen, Germany • N. Kuklik: Institute for Medical Informatics, Biometry and Epidemiology, University Hospital Essen, Germany; Centre for Clinical Trials Essen, University Hospital Essen, Germany • G. Metzner: Section of Health Care Research and Rehabilitation Research, Institute of Medical Biometry and Statistics, Faculty of Medicine and Medical Center, University of Freiburg, Germany • N. Mueller: Knappschaftskrankenhaus Bottrop GmbH, Academic teaching hospital of the University of Duisburg-Essen, Germany • L. Seiler: Institute of Technical Chemistry, Leibniz University Hanover, Germany; KfH Foundation for Preventive Medicine, Germany • S. Stolpe: Institute for Medical Informatics, Biometry and Epidemiology, University Hospital Essen, Germany • C. Blume: Institute of Technical Chemistry, Leibniz University Hanover, Germany; KfH Foundation for Preventive Medicine, Germany
Name and contact information for the trial sponsor {5b}	Study leader: Prof. Dr. Cornelia Blume (blume@iftc.uni-hannover.de)
Role of sponsor {5c}	This is an investigator-initiated trial. The study leader designed the funding application. All German statutory health insurances contribute to the Innovation Funds and the health insurance Knappschaft-Bahn-See provides the hospital information system (HIS). The funder played no role in designing the study and will not be involved in the data collection, analysis, interpretation of data, writing the report, or the decision to submit for publication. The funder takes part in the administrative management of the study as a controlling authority and reviews the results of the study with the purpose of deciding (together with representatives of the health insurance companies) whether the new form of health care shall be prospectively included in the fee schedule for billable health care services in Germany.

## Introduction

### Background and rationale {6a}

The prevalence of impaired renal function in the general population aged 45 years and older is about 10%. It is significantly higher in persons with hypertension, people with diabetes and those older than 70 years [1]. Chronic kidney disease (CKD), subsequent cardiovascular events and renal replacement therapies place a disproportionate burden on the healthcare system [2]. Early identification of CKD patients with advanced or rapidly progressive renal insufficiency and consistent specialist care can slow disease progression [3–5]: Risk factors for the progression of CKD, such as hypertension, diabetes and inadequate medication, can be better controlled with early specialist care. The incidence of medical complications and the resulting need for frequent hospitalisations are significantly reduced. Currently, no systematic CKD management has been established in Germany. A treatment guideline on the care of non-dialysis-dependent CKD patients in general practice was published in 2019 [https://www.awmf.org/leitlinien/detail/ll/053-048.html], but awareness of the importance and potential consequences of chronic renal disease is low among physicians and patients [6]. Because renal insufficiency can be asymptomatic even at higher stages, it is often unknown even in those from high-risk groups [7, 8]. Even when renal insufficiency is known, risk factors such as hypertension and diabetes are not adequately controlled [9, 10].

Identifying patients in need of specialised CKD management is therefore relevant both from a patient perspective and for economic reasons. Because CKD is a common comorbidity in hospitalised patients, cross-sector collaboration is useful in this setting to provide needs-based care for CKD patients. Structured referrals of hospitalised patients to the outpatient sector have resulted in improvements in various endpoints (mortality, rehospitalisation) for several conditions [11]. Increased attention to hospitalised patients with renal insufficiency and their referral to specialised care after discharge reduced mortality and the risk of end-stage renal disease (ESRD) in a Belgian study [12]. The German guideline for the care of non-dialysis CKD in general practice recommends referral of a CKD patient to a specialist when CKD is first diagnosed with an estimated glomerular filtration rate (eGFR) according to CKD epi of < 30 ml/min or with an eGFR of ≤ 60 ml/min with concomitant presence of albuminuria stage ≥ A2 (= albumin-creatinine ratio 30–300 mg/g), among others. The extent to which these recommendations are implemented for CKD patients in the context of primary care is not known. Studies on the referral practice of CKD patients have shown that, in addition to general practitioners’ lack of knowledge about CKD management and guidelines, the patient's attitude towards their disease can also lead to delayed referral to a specialist [13].

This study aims to clarify whether, for hospitalised patients with advanced chronic renal disease (specifically, with a Kidney Failure Risk Equation [KFRE] score of 9% to require dialysis within the next 5 years), a risk-based appointment with a nephrology specialist reduces disease progression. The KFRE score calculation was developed in independent Canadian patient cohorts in 2011 and validated internationally [14]. In studies of optimal care for CKD patients, the estimated 5-year ESRD risk has already been used for risk-based stratification of CKD patients for referral to a specialist in non-German populations [15, 16]. In its most commonly used form, it requires patient data on age, sex, eGFR and albumin/creatinine ratio (ACR). A formula without the use of ACR is also available and has been developed for validation for Dutch CKD patients [17].

The change in estimated glomerular filtration rate (eGFR) and control of risk factors such as hypertension and HbA1c in diabetic patients are used for assessment. Two groups of patients will be compared, one with the intervention of an appointment with a nephrologist, and one without this appointment in further routine care.

## Objectives {7}

The main objective of the study is to assess if risk-based discharge management related to the predicted 5-year risk of end-stage renal disease leads to needs-based specialist care for chronic renal disease and reduces further decline in renal function in terms of eGFR. Additional hypotheses are that the proportion of affected patients with uncontrolled hypertension—and/or uncontrolled HbA1c in diabetics—is reduced, and likewise, the medication that is not in line with guidelines or inadequate concerning renal function. Furthermore, the evaluation will include economic considerations and interviews with contributing physicians to assess the acceptance and feasibility of the intervention.

## Trial design {8}

The study is conducted in a stepped-wedge cluster-randomised design with 8 clusters, i.e. medical centres, after initially planning with 4 clusters (see Sample size section). All centres start with the recruitment of patients for the control group. After a time lag determined by randomisation in the stepped-wedge design, each cluster switches to the intervention phase. Ideally, equal numbers of patients should be recruited for control and intervention groups. The study aims to confirm the superiority of risk-based appointment management in CKD patients compared to the usual discharge management.

**Fig. 1:**
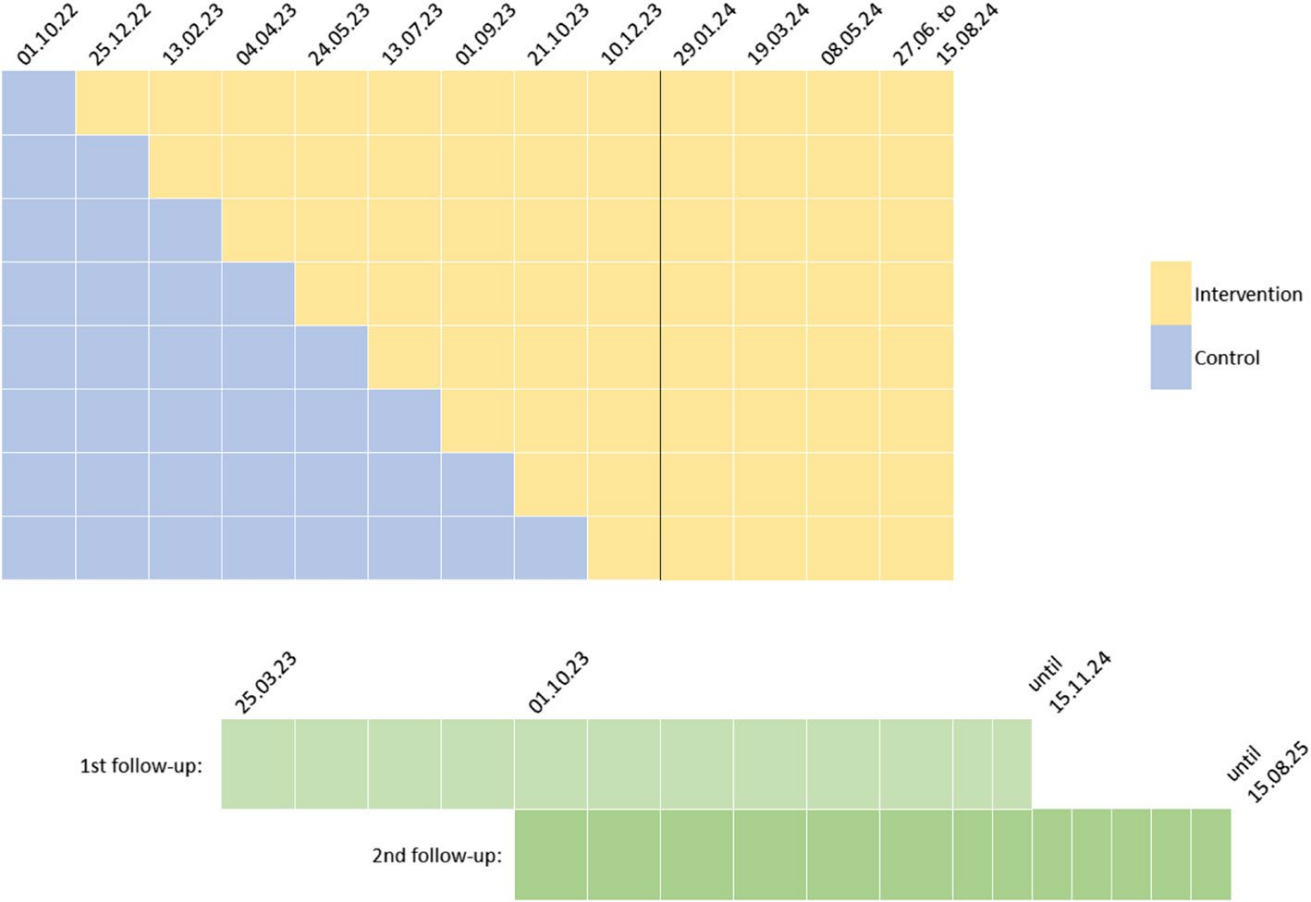
Modified stepped-wedge design of the MinDial study, depicting phases of recruiting into the control and intervention groups, respectively, and the follow-up schedule. In the stepped-wedge illustration, each row represents one of the eight medical centres (clusters). Four additional phases were incorporated later due to underrecruitment in the intervention group. The first follow-up applies only to patients in the intervention group

The stepped-wedge design and recruitment schedule are depicted in Fig. 1. Originally, nine recruitment phases of 50 days each were planned. In November 2022, the first recruitment phase (of control patients) was extended by 5 weeks to compensate for a slow start. Moreover, because of underrecruitment especially regarding the intervention group, it was decided in October 2023 to extend the recruitment by four additional phases.

## Methods: participants, interventions and outcomes

### Study setting {9}

Patient recruitment takes place in North Rhine-Westphalia, Germany, in four hospitals of the miners’ health insurance company (’Knappschaft’) with a total of eight centres: Dortmund, Bottrop, Marl, Lünen, Kamen, Gelsenkirchen, Recklinghausen and Lütgendortmund. The hospitals are academic teaching hospitals of the University of Duisburg-Essen and the Ruhr University Bochum.

### Eligibility criteria {10}

#### Inclusion criteria

- Age ≥ 18 years
- Admitted to one of the participating hospitals
- Health insured with ‘Knappschaft-Bahn-See’
- Existing chronic renal disease (either already known and documented in the hospital information system as diagnoses ICD-10 N04.9, N17.99, N18.1–9, N19, R31, R80, or identified during hospitalisation (as distinct from acute renal failure))
- Risk of end-stage renal disease within 5 years according to KFRE score of at least 15% (date of laboratory determination before 20 June 2023) or at least 9% (since 20 June 2023), respectively; see the section ‘Participant timeline {13}’ for details on this change.
- Consent to contacting the general practitioner (GP) and specialists after discharge
- General state of health sufficient for study participation

#### Exclusion criteria

- Pregnancy
- Patients with ≥ 2 consultations with a nephrology specialist in the last 12 months before study inclusion
- Inclusion in the MinDial study during a previous hospitalisation

### Who will take informed consent? {26a}

The study nurses inform eligible patients about the study and ask them if they consent to participate. If a patient has questions that cannot be answered by the study nurse, a study physician will be consulted before confirming the consent forms with the patient’s signature.

### Additional consent provisions for collection and use of participant data and biological specimens {26b}

In the informed consent form, participants may optionally consent to provide their health insurance data for further assessment following the study.

### Interventions

#### Explanation for the choice of comparators {6b}

The control group receives standard care, i.e. patients are discharged from the hospital as usual. This is the comparator of choice since the aim of the study is to assess if a risk-based appointment with a nephrology specialist reduces disease progression compared to current routine care.

#### Intervention description {11a}

The intervention consists of making an appointment with a nephrology specialist as part of hospital discharge management for patients in the intervention group. Control patients will not receive a study-related intervention. In detail, the intervention is organised as follows: A nephrology study nurse selects three nephrologists near the patient’s home. The patient selects a specialist from this list, with whom an appointment is then scheduled within the next three months after discharge. The study nurse at the respective site then passes the appointment on to the patient.

#### Criteria for discontinuing or modifying allocated interventions {11b}

If a study patient turns out to suffer from acute instead of chronic renal insufficiency, the intervention is cancelled for this patient. These patients are excluded from further follow-up.

#### Strategies to improve adherence to interventions {11c}

One week after the agreed appointment, the study nurse calls the intervention patients to ask whether they have kept their appointment with the nephrologist and, if not, whether the nurse should arrange a new appointment.

#### Relevant concomitant care permitted or prohibited during the trial {11d}

Any concomitant treatments and measures are permitted during the trial.

#### Provisions for post-trial care {30}

No provisions are made for ancillary care, post-trial care or compensation. Patients in the control group are treated as in routine care and it can be expected that the intervention, consisting of an appointment with a nephrologist, will not be detrimental to the patient.

### Outcomes {12}

#### Primary endpoint

- Difference in mean change in eGFR in ml/min/1.73 m^2^ (after chronic kidney disease epidemiology collaboration [CKD-EPI]) between the intervention and control group from baseline to 12 months after hospital discharge

The eGFR was chosen as the primary parameter because it is considered a good indicator for the assessment of kidney patients and has also been used in numerous nephrology studies over time [18].

#### Secondary endpoints

Differences between intervention and control group, 12 months after hospital discharge:

- Difference in proportion (%) of patients with controlled blood pressure (≤ 140/90 mmHG)
- For diabetics: difference in proportion (%) with guideline-compliant HbA1c (6.5 to ≤ 7.5%)
- Difference in proportion (%) of patients with non-guideline-compliant care about the prescription of selected medication groups (including statins, RAS blockers, ARBs and ACEIs; based on pre-specified ATC codes classified as non-guideline-compliant) according to the assessment of medication plans by consultant nephrologists
- Difference in the proportion (%) of patients with non-guideline-compliant medication (resulting from the aforementioned assessment of medication plans by several consultant nephrologists)
- Difference in the mortality rate (%)

#### Health economics

Intervention costs and costs of medical service utilisation in relation to the primary and secondary endpoints based on cost-effectiveness analyses

#### Process quality, acceptance and feasibility

- Comparison between intervention and control group:
  Difference in proportion (%) with a nephrology specialist consultation at 3 months from discharge
- Intervention patients:
  Perceived quality of care (Patient Reported Outcome [PRO], 3 + 12 months), after 3 months incl. factors from the patient’s perspective that led to non-utilisation of nephrology care
  Perception of illness (PRO: Brief-Illness-Perception-Questionnaire [BIPQ], 3 + 12 months)
  Knowledge about kidney disease (PRO: Perceived Kidney Knowledge Survey [PIKS], 3 + 12 months)
  Health-related quality of life (PRO: Short Form 12 [SF12], 3 + 12 months)
- Control patients:
  Perception of illness (PRO: BIPQ, 12 months)
  Knowledge about the kidney disease (PRO: PIKS, 12 months)
  Health-related quality of life (PRO: SF12, 12 months)
- GPs/specialists:
  Acceptance and feasibility of this new form of care (risk-based appointment scheduling): Semi-structured interviews with specialists (*N* = 4, 30 min) and GPs (*N* = 8, 20 min)

### Participant timeline {13}

#### Screening

For newly admitted patients, the information on the patient in the hospital information system (HIS) is checked for known concomitant renal disease (ICD10 codes: N04.9, N17.99, N18.1-0.9, N19, R31, R80) or newly detected impaired renal function (eGFR < 60 ml/min/1.73 m^2^). If chronic renal disease is indicated, the patient’s 5-year ESRD risk will be estimated using the KFRE formula.

#### Evaluation of eligibility

**Fig. 2:**
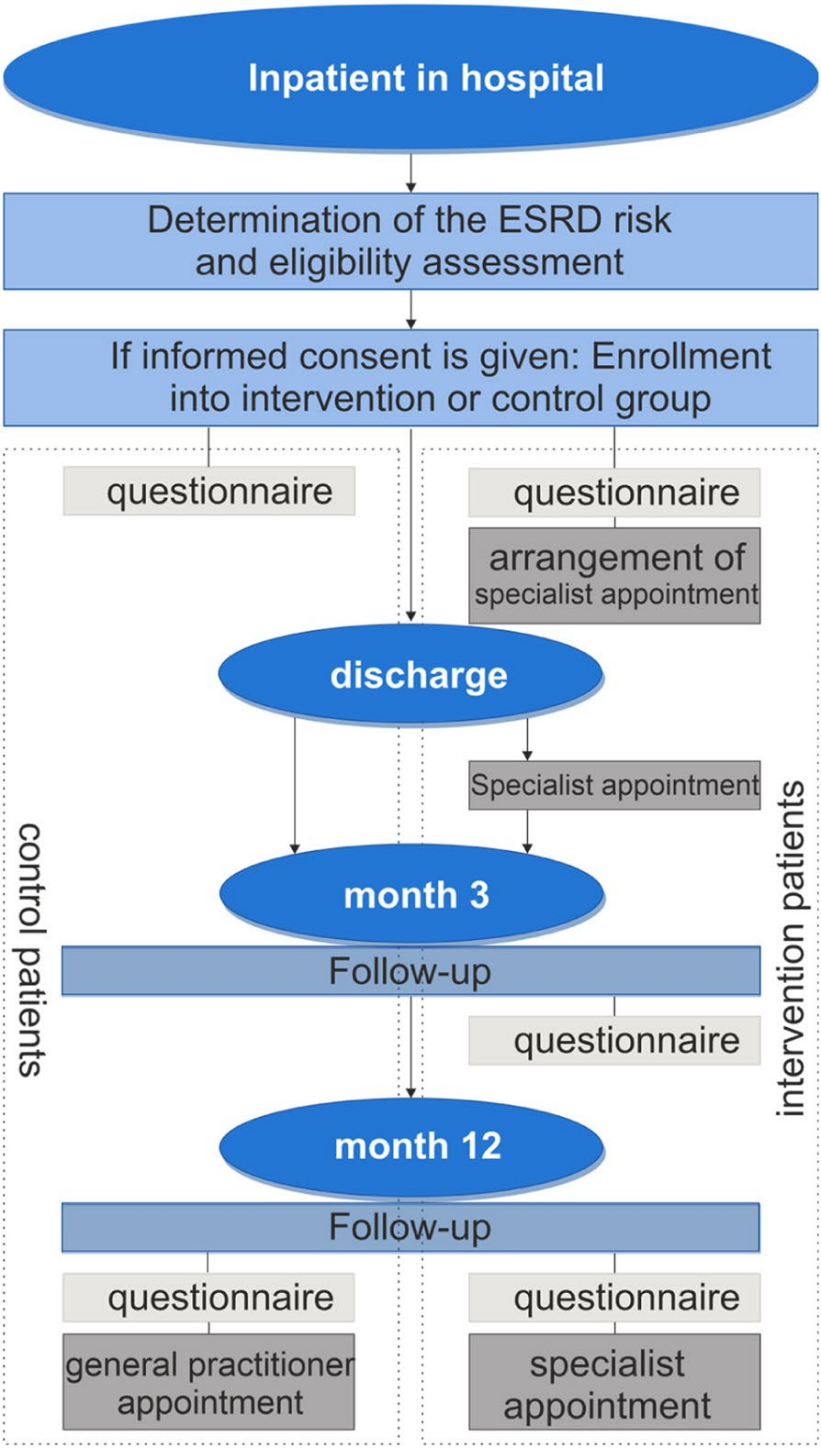
Patient enrolment and follow-up in the MinDial study. Generally, eligibility is assessed by the study nurses. In doubtful cases, the decision for or against inclusion is made by study physicians, as detailed in this section

If a study patient meets all inclusion criteria and none of the exclusion criteria, this is indicated to the corresponding study nurse in the HIS. In the first step, the study nurse classifies this patient (Fig. 2), assessing whether the patient has chronic renal disease based on the available patient data and entries in the HIS. If chronic renal disease appears certain, the patient will be informed about the study and asked for consent.

#### Consultation (study physicians)

If the study nurse cannot determine a definite chronic renal disease or study eligibility appears questionable due to critical health conditions (infirm prognosis, the patient may not survive the study), the electronic medical record will be forwarded to a study physician for evaluation. Therefore, a special mask implemented for this study in HIS is used, where the study nurses can tag a patient and additionally leave a note with the emerged question. The next time the patient is checked by the study doctor, he can immediately see the marked patients, process the requests and mark the patients back for the study nurses.

#### Recruitment (or exclusion from the study)

After a positive assessment of the patient by the study nurse or study physician, the informed consent form and an initial questionnaire are printed for the corresponding patient. After the study nurse has informed the patient about the study and the patient or the legal guardian has consented to the study participation, the patient is moved to the category ‘has consented’ in the HIS, and the informed consent form, as well as the initial questionnaire, is scanned and stored in the HIS.

The exclusion of patients from the study is documented with corresponding reasons.

#### Baseline data

Once a patient is recruited for the study, he or she is entered into an electronic case report form (eCRF, Clincase). The patient receives an identification number, the so-called Clincase key. This key is also used for subsequent pseudonymised merging with possible additional health insurance data, which can be coded with an insurance key. Subsequently, the study-relevant patient data are transferred to the eCRF (details on the data collection are depicted in Table 1).

**Table 1:** Variables of interest and time of collection

Variable	Baseline	3-month FU Intervention	12-month FU Patient	12-month FU Physician	Category
Age (QN/HIS)	X				BL
Sex (QN/HIS)	X				BL
Informed consent date (QN)	X				Orga
‘People of Color’ membership	X				BL
Signatory (patient/caregiver)	X				Orga
Consent healthcare data (QN)	X				
Consent subsequent contact (QN)	X				IC
General practitioner details (HIS)	X				Orga
Study arm (HIS)	X				
Date of QN + interview	X	X	X		BL
**Patient characteristics (QN)**					
Weight	X				BL
Height	X				BL
Education level	X				BL
Living situation	X				BL
Smoking status	X				BL
General health condition	X				BL
Health literacy	X				BL
**Known diagnoses (HIS)**					
Chronic kidney disease	X				BL
Hypertension	X				BL
Diabetes	X				BL
Heart attack	X				BL
Charlson Index	X				BL
**Lab values (HIS)**					
Serum creatinine	X			X	PE
eGFR	X			X	PE
KFRE score	X				IC
HbA1c/glycosylated hemoglobin	X			X	BL | SE
Blood pressure (diast./syst.)	X			X	BL | SE
**Assessment of medication at discharge (HIS)**					
Guideline compliance	X				BL
Antihypertensives count	X				BL
ACEI/ARB/sartans	X				BL
SGLT2 inhibitors	X				BL
Statins	X				BL
Nephrotoxic medication	X				BL
**Assessment of medication at 12-month FU (GP/specialist)**					
Guideline compliance				X	SE
Antihypertensives count				X	SE
ACEI/ARB/sartans				X	SE
SGLT2-inhibitors				X	SE
Statins				X	SE
Nephrotoxic medication				X	SE
Vital status				X	SE
New onset diabetes mellitus				X	SE
**Medication costs (GP/specialist)**					
Name of drug/active ingredient/dosage/daily dose				X	HE
**Evaluation of intervention by patient (QN)**					
Appointment attended		X			Pr
Reasons for non-attendance		X			Pr
New appointment		X			Pr
Evaluation of appointment scheduling		X			Pr
Evaluation of consultation		X			Pr
Overall evaluation		X			Pr
**Evaluation of intervention by GP/specialist**					
GP (interview)				X	Pr
Nephr. specialist (interview)				X	Pr
**Utilization (QN)**				X	
Number of GP visits		X	X	X	HE
Number of specialist visits with date		X	X	X	HE
**PROs for process evaluation (QN)**					
Knowledge about kidney disease (PIKS)		X	X		Pr
Health-related quality of life (SF12)		X	X		Pr
Perception of illness (BIPQ)		X	X		Pr
**Costs of intervention (study nurse)**					
Working hours of study nurse	X	X			HE

*Abbreviations: ACEI* angiotensin-converting enzyme inhibitor, *ARB* angiotensin-receptor blockers, *BIPQ* Brief Illness Perception Questionnaire, *BL* baseline, *eGFR* estimated glomerular filtration rate, *FU* follow-up, *GP* general practitioner, *HE* health economics, *HIS* hospital information system, *IC* inclusion criterion, *KFRE* kidney failure risk equation, *Orga* organisation, *PE* primary endpoint, *PIKS* Perceived Kidney Knowledge Survey, *Pr* process evaluation, *PRO* patient-reported outcome, *QN* questionnaire, *SE* secondary endpoint, *SF12* Short Form 12, *SGLT2* sodium-glucose co-transporter 2

To document serum creatinine, KFRE score, blood pressure and HbA1c in the eCRF, all values are taken at a time interval around the date when the KFRE score is ≥ 15% (date of laboratory determination before 20 June 2023) or ≥ 9% (since 20 June 2023) for the first time in the current case.

#### Follow-up

##### Three-month follow-up after discharge (intervention patients)

Three months after discharge, intervention patients are contacted by mail and asked to fill out a questionnaire. Patients are asked whether they utilised the appointment by the nephrologist and if yes, how they assess the benefit of this referral. In addition, their quality of life (SF12), the knowledge about their kidney disease (PIKS), and how they perceive their disease (BIPQ) are asked via standardised questionnaires.

##### Twelve-month follow-up (all patients)

One year after discharge from the hospital, a follow-up is performed for each patient regarding renal function, HbA1c, blood pressure, medication, frequency of physician visits, quality of life, knowledge about the disease and illness perception. For this purpose, the GP and/or nephrologist are contacted by the study nurse by mail and/or phone to check the vital status of the patient and to request laboratory data concerning serum creatinine and Hba1c in diabetic patients, as well as the latest three blood pressure measurements, the number of patients visits during the last year, incident diabetes and the actual medication (via the standardised medication plan). The medication is assessed whether relevant medication classes are included (antihypertensives, statins, ACE inhibitors or RAAS blockers, and SGLT2).

The data collected at the 3-month and 12-month follow-ups are detailed in Table 1, together with the baseline data.

#### Questionnaires

In principle, the questionnaires are to be answered by the study participants themselves. If this is not possible for elderly or mentally incompetent patients, the legal guardian can answer the questionnaires for the patient. It is documented whether the answers were given exclusively by the caregiver/relatives or by the patients themselves.

### Sample size {14}

#### Original sample size calculation (before shifting from 4 to 8 clusters)

The sample size calculation was based on the primary endpoint. If after 12 months there is a mean difference in the decline of eGFR of 2 ± 4 ml/min/1.73 m^2^ (according to CKD-EPI; Cohen’s *d* = 0.5) between the control and intervention groups, in the cross-sectional complete cluster-randomised design chosen here (4 clusters, 4 steps, 5 measurement time points/periods), a difference can be detected in a linear mixed model with the Wald *z*-test for equality of means at the two-sided significance level *α* = 5% with a power of 80%, when 340 observations are available (PASS, version 15.0.3). Here, an intra-cluster correlation coefficient of 0.02 was assumed; this magnitude seems realistic, since the intervention is not directed at the treating physicians in the hospitals, but at the patients [19]. Furthermore, the assumed standard deviation of σ = 4 ml/min/1.73 m^2^ (assuming an eGFR variability [20] of 10% of the maximum eGFR of 40 ml/min/1.73 m^2^ expected in the patient population) seems realistic, and the mean difference of 2 ml/min/1.73 m^2^ seems achievable as well as clinically relevant [4]. Since we anticipate a drop-out of 20% at 12 months follow-up, *n* = 425 patients should be enrolled in the study, 85 in each of the 3-month periods until the next change of a cluster to the intervention phase.

#### Updated power calculation for the stepped-wedge design with 8 clusters

Before the start of the project, it was assumed that the shift from control to intervention conditions would have to take place simultaneously at all sites of a hospital (each with two sites) and that a design with four clusters would therefore have to be used. As it became clear that the eight sites were independent of each other in this respect, a design with one cluster per site was used instead, to improve the statistical power with the same use of resources.

An analysis of hospital data from 2020/21 after the project started had shown that recruitment numbers were expected to vary widely between sites in some cases. Assuming that 340 patients with complete follow-up would be recruited as planned, the following distribution among the 8 sites could be expected: 77, 63, 50, 37, 37, 34, 25 and 17 participants. By switching to a design with 8 clusters (one cluster per site) and under otherwise unchanged assumptions for the sample size calculation, a power of 87.5% was now expected before randomisation (R version 4.1.3, CRTpowerdist version 0.4.0 [21]). Since the actual power depends on the randomisation result, randomisation was performed under the constraint of largely balanced expected intervention and control group sizes. For this purpose, the set of possible randomisation outcomes was restricted to those where a maximum size difference between the intervention and control group of 20 subjects was expected. This corresponded to the exclusion of the less balanced half of all theoretically possible randomisations.

#### Second update of the power calculation (based on interim sample size)

In the fourth quarter of 2023, the statistical power was recalculated. In contrast to the previous calculations, actual recruitment figures were taken into account for each site and recruitment period (for periods 1–7; projections based on past recruitment numbers were used for periods 8–13). For an optimistic prediction of future recruitment numbers, the mean values of the three stronger recruitment periods were assumed. According to this prediction and factoring in a 20% drop-out rate, 286 patients with complete follow-up are expected to be available for the final analysis.

Considering the slow recruitment, it was decided to switch to one-sided testing to increase statistical power. In the original planning, impartial two-sided testing was planned, which would have allowed verification of both an advantage and a disadvantage of the intervention. On the one hand, it is much more relevant in the context of health care to prove an advantage of the intervention than a disadvantage. On the other hand, it is more likely to assume an advantage (of unknown magnitude) than patients getting worse in terms of their kidney function as a result of an appointment with a nephrologist.

With one-sided testing and otherwise unchanged assumptions (regarding effect size, intra-cluster correlation coefficient and significance level), this corresponds to a power of 72.1% (R version 4.3.2, swCRTdesign version 4.0 [22]). The power would be 80.0% if the actual mean difference in the decrease in eGFR is 2.23 ml/min/1.73 m^2^ instead of the originally assumed 2.0 ml/min/1.73 m^2^.

### Recruitment {15}

Hospital data from the past years (2020, 2021) was used to examine the potential of each of the eight medical centres to enrol eligible participants. Achieving the target sample size of *n* = 425 participants based on this data seemed realistic. Moreover, the study schedule included an optional time slot to extend the recruitment beyond 15 months, if necessary.

In November 2022, the first recruitment phase (of control patients) was prolonged by 5 weeks to compensate for a slow start. Because of ongoing underrecruitment especially regarding the intervention group, in October 2023, it was decided to extend the recruitment by four additional phases, 50 days each.

## Assignment of interventions: allocation

### Sequence generation {16a}

According to the cluster-randomised stepped-wedge design, the eight participating medical centres were randomised to eight intervention start times. Randomisation was carried out under the constraint of roughly balanced expected intervention and control group sizes, considering the anticipated recruitment potentials of each medical centres. The allocation sequence was generated by a computer, using a self-written R program and the date as random seed (‘20,220,727’).

### Concealment mechanism {16b}

The randomisation list was concealed from the participating study sites. After randomisation, each medical centre was informed about when it would enter the intervention phase. All switch dates are known to the study group. Subsequent allocation of study participants to the control and intervention groups according to the switch dates is not concealed.

### Implementation {16c}

Randomisation of clusters was performed at the Institute of Medical Biometry and Statistics, Faculty of Medicine and Medical Center – University of Freiburg, Germany. Participants are enrolled by study nurses. Allocation to the intervention or control group is determined by the stepped-wedge design: At the beginning of the study, all sites recruited into the control group. After the first period, one site switched to intervention conditions. At intervals of 50 days, clusters 2 to 8 also switch to the intervention phase.

## Assignment of interventions: blinding

### Who will be blinded {17a}

Due to the study design and the type of intervention, no blinding is possible.

### Procedure for unblinding if needed {17b}

Not applicable, since no blinding is used.

## Data collection and management

### Plans for assessment and collection of outcomes {18a}

#### Baseline

Data on laboratory measurements (serum creatinine and HbA1c), blood pressure, pre-existing illnesses and medication are taken from the patient’s medical record in the hospital information system (HIS). Additional data on demography, height and weight, smoking status and housing as well as self-assessed health status and health literacy is collected by questionnaire during the hospital stay. Only blood analysis values from the respective laboratory at each site are used. Serum creatinine is measured according to Jaffé.

#### Follow-up at 3 months

Patients in the intervention group receive a mailed questionnaire and are asked to state whether they attended the appointment by the nephrologist and how they rate its benefit. Additionally, the standard questionnaires SF12, PIKS and B-IPQ are used for the patient-reported outcomes (PRO).

#### Follow-up at 12 months

All study patients are asked about their perceived quality of care, their perception of the disease (B-IPQ), their level of information about the disease (PIKS) and their quality of life (SF12).

Follow-up data on laboratory measurements, incident diabetes and medication is collected from the GPs and/or nephrologists by post/fax/email. Physicians receive financial compensation for their efforts.

#### Acceptance and feasibility

Interviews with 8 GPs and 4 nephrologists with their assessments of the risk-based appointment system for hospital patients are recorded and analysed.

### Plans to promote participant retention and complete follow-up {18b}

The recruited patients in the intervention group are reminded of their appointment with the nephrologist and are offered to get a second appointment if they have not been able to attend the initially organised visit. About 11 months after hospital discharge, all patients are asked by postal mail to make an appointment with their GPs or nephrologists if possible. This shall ensure that primary and secondary endpoint information will be available. The GPs and nephrologists consulted are initially and repeatedly informed about the course of the study. These medical colleagues will receive an expense allowance for participating in the study according to the current fee schedule for physicians, e.g. for providing data. If the follow-up questionnaire is not returned by a physician within a month, they will be contacted by telephone and the documents may be sent again by post or fax. Participants are equally contacted by phone if they do not return the follow-up questionnaire in time. The questionnaire then can be administered by telephone.

### Data management {19}

The study-related data from the HIS, the baseline patient questionnaire, the follow-up questionnaires and the information from the GP and specialist at the 12-month follow-up are recorded in the eCRF (Clincase). The data is entered by trained study nurses. Access to the data entry screens is password-protected. The eCRF includes an audit trail to document any data changes.

Data plausibility is checked on data entry into the eCRF with automatically generated queries. Additionally, more advanced data checks are programmed in SAS 9.4 (SAS Inc. Cary, NC, USA) and are run on a weekly basis. Resulting queries are returned to the study nurses for completion.

### Confidentiality {27}

The data is stored in the eCRF software Clincase. Study documents (questionnaires, information sheets, data collection sheets in exchange with the physicians in the follow-up) are scanned and stored in the HIS. The original sheets are stored in locked cabinets. Only the study nurses and the study doctors have access to patient data in the HIS and to the cabinets.

### Plans for collection, laboratory evaluation and storage of biological specimens for genetic or molecular analysis in this trial/future use {33}

Not applicable; no biological specimens are collected for genetic or molecular analysis.

## Statistical methods

### Statistical methods for primary and secondary outcomes {20a}

In the confirmatory primary analysis, the effect of the intervention on the primary endpoint (decrease in eGFR at 12 months) is tested in a linear mixed model, estimated and reported with a one-sided 95% confidence interval ([23]). The model includes the intervention (with versus without modified discharge management), the categorical periods as fixed effects, the baseline measure of eGFR, age at study inclusion and gender as covariates. Intra-cluster variation, i.e. membership of a cluster, is modelled via random intercept. The one-sided test to prove the benefit of the intervention is performed based on the respective one-sided 95% confidence interval from the linear mixed model. Since the follow-up examination will not always take place exactly 12 months after discharge, the decrease in eGFR from the baseline to the follow-up measurement (i.e. the dependent variable in the regression model) is proportionally converted to a 12-month difference.

Intervention effects for binary secondary outcomes will be estimated and tested similarly, using appropriate generalised linear mixed models.

#### Health economic evaluation

The health economic evaluation examines the relationship between the additional costs and the additional benefits of the intervention using classic cost-effectiveness analyses. The short-run costs of the intervention (calculation of the 5-year risk of kidney failure and making an appointment with a specialist) are derived from the documented workload of the nursing staff involved. The costs of the medication during the 12-month follow-up are approximated from the medication plan at 12 months after discharge. Lastly, the costs of GP and specialist visits during the 12-month follow-up are calculated based on standardised unit costs [24].

After summarizing these costs on the patient level, a cost-cost comparison between the intervention and control group is carried out using linear mixed models, analogously to the analysis scheme described above. Finally, the additional costs of the intervention derived from this are compared with the results of the analyses regarding the primary and secondary endpoints. For inference, a 95% confidence interval is calculated for the individual cost-effectiveness ratios, e.g. for the additional costs per eGFR improvement, using Fieller's theorem ([25, 26]).

#### Process quality

The percentage of patients with a nephrology specialist consultation within 3 months of discharge in both groups is compared descriptively. The perceived quality of care, reasons for not consulting a nephrologist, subjective concept of illness, knowledge of kidney disease and health-related quality of life in the intervention patients group are also presented descriptively. Subjective disease concept, knowledge about kidney disease and health-related quality of life 12 months after discharge are compared between the intervention and control groups.

#### Acceptance and feasibility

To evaluate the acceptance and feasibility of the new intervention, the risk-based appointment scheduling, semi-structured interviews with the participating nephrologists and GPs will be conducted. With individual interviews, we aim to explore the nephrologists and GPs appraisal of the intervention. Therefore, interview guidelines will be developed for each of the two groups, starting with an open narrative question followed by questions concerning the themes of interest. These include the doctors’ experiences with the new intervention, the appraisal of individually perceived benefits and costs of the intervention, the cost–benefit ratio, possible improvements in the collaboration between the different care sectors (hospital, nephrology specialists, GPs) through the intervention, perceived feasibility in routine care and ideas for optimizing the intervention. Parts of the interview guidelines for the nephrologists and GPs will be the same to obtain the different perspectives on the risk-based appointment scheduling. In addition, the interview guidelines will consist of specific questions for the two groups. We aim to interview *N* = 4 nephrologists and *N* = 8 GPs. A purposeful sampling method will be used to achieve maximum variation within the sample, as intended in qualitative research. Pre-defined selection criteria such as age, gender, work experience, or urban vs. rural region of the location of the practice will be considered. The doctors should take care of at least one study patient. A trained project research associate who is not involved in the intervention provision will conduct the interviews. The interviews will take place in parallel with the intervention phase. It is estimated that the interviews with the nephrologists will last about 30 min and the interviews with the GPs 20 min. The interviews will be audio-recorded and transcribed verbatim. The transcribed interviews will be analysed following qualitative content analysis (cf. [27]). In this method of content structuring, the data material is categorised and coded in an iterative process using an inductive and deductive approach. To ensure a transparent, replicable analysis procedure, coding rules with anchor examples will be documented. The Theoretical Framework of Acceptability (TFA; [28]) will guide the development of the interview guidelines and the analysis.

### Interim analyses {21b}

Since the intervention consists of providing an appointment with a nephrology specialist as part of hospital discharge management and the control patients receive no study-related intervention, there is no need for interim analyses concerning a possible early termination of the trial. However, during recruitment and follow-up, analyses are performed concerning the data quality.

### Methods for additional analyses (e.g. subgroup analyses) {20b}

Since the cut-off value to identify patients with an increased risk of end-stage renal disease within 5 years was changed from 15 to 9% during the recruitment period (see the section ‘Participant timeline {13}’ for details), an additional subgroup analysis will be performed, focusing on the originally planned collective of patients with risk score ≥ 15%.

### Methods in analysis to handle protocol non-adherence and any statistical methods to handle missing data {20c}

Participants are analysed ‘as randomised’ (being cluster-randomised instead of randomised individually), independent of their medical treatment after hospital discharge. Participants without 12-month follow-up are reported in a CONSORT flowchart. Missing data of these participants are accounted for in the evaluation under the missing at random assumption.

### Plans to give access to the full protocol, participant-level data and statistical code {31c}

This paper provides most of the content of the internal study protocol. Currently, there are no plans to grant public access to code or participant-level data (for data protection reasons).

## Oversight and monitoring

### Composition of the coordinating centre and trial steering committee {5d}

The MinDial study group comprises teams located in various cities in Germany, responsible for different subject matters:

- Study lead and management
- Study physicians in the participating medical centres
- IT team for developing risk score estimating tool and integration with the hospital information system
- Study nurses (informing and including the patients, conducting the intervention, collection and entering data, organisation of follow-up of patients, contacting physicians)
- Data management team (plausibility checks and data quality visits) and study software surveillance
- Statisticians and healthcare researchers for study evaluation

Meetings within teams take place regularly, and meetings of the whole study group are scheduled following the roadmap (and as needed).

### Composition of the data monitoring committee, its role and reporting structure {21a}

A data monitoring committee is not installed. It is not to be expected that the intervention in MinDial can cause any harm to the participants, as it should enhance routine care. Therefore, it is not necessary to monitor data to assess potential safety risks for the participants. Considerations of whether any changes in the protocol are necessary to enhance participants’ adherence are made by the study lead and coordinator in cooperation with the evaluation team and the epidemiologic advisories.

### Adverse event reporting and harms {22}

Not applicable due to the type of intervention.

### Frequency and plans for auditing trial conduct {23}

No on-site auditing of trial conduct is planned. Formal auditing is handled by the German Space Agency (DLR), commissioned by the funder. The DLR expects interim reports concerning the adherence to the roadmap and does the financial controlling.

### Plans for communicating important protocol amendments to relevant parties (e.g. trial participants, ethical committees) {25}

The study leaders meet regularly. The coordinator points out relevant and necessary changes to the protocol in these meetings. IMIBE advises the study leaders on these protocol changes after careful discussion within the study centre of the University of Duisburg-Essen, taking into account the applicable study regulations. Data protection issues are discussed with the legal advisor and the responsible data protection officers in the clinics.

## Dissemination plans {31a}

- Contribution to nephrological and/or epidemiological publication organs
- Referral to the German Nephrological Society (Congress) or to the Congress of the American Society of Nephrology
- Referral to the Knappschaftskliniken and to the Knappschaftsversicherung
- Publication of the final report of the MinDial study group by the Federal Joint Committee (G-BA)

## Discussion

Since the start of participant recruitment, new insights were gained and several issues needed to be addressed. For instance, the composition of study participants differed noticeably from the intended target population, especially with regard to their age distribution. Hence, it was decided to decrease the cut-off value to identify patients with an increased risk of end-stage renal disease within 5 years from 15 to 9%. In general, recruitment is slower than expected. Beyond the adjusted cut-off value, counter-measures include extending the recruitment period by half a year, refreshing the training for the study nurses and improving the enrolment process. Since the recruitment target still had to be corrected downwards, two-sided testing was replaced by one-sided testing in the statistical analysis plan to increase power. It was also found that ruling out acute kidney failure is more time-consuming than anticipated. Potential study participants are sometimes not in the hospital long enough to be approached and enrolled after acute kidney failure has been ruled out. However, while this makes recruitment more challenging, it would not be a problem in routine care.

## Trial status

Patient recruitment started on 01 October 2022 and will end on 15 August 2024, according to plan. The current study protocol is Version 3 from November 2023. 

## Data Availability

Data access is regulated according to a formal data protection concept, which was part of the application for ethics approval, and limited to those authorised to access the data. Technical and organisational measures for data protection and data security apply at the Institute for Medical Informatics, Biometry and Epidemiology (IMIBE) of the University Hospital Essen, and for the web mailer of the Knappschafts-Bahn-See insurance. For evaluation, the final anonymised dataset is provided by the IMIBE for the Institute of Medical Biometry and Statistics (IMBI) at the University of Freiburg. It is stored on protected drives at the IMIBE and IMBI. The study directors and the IMIBE team will have further access.

## Abbreviations

ACE: Angiotensin-converting enzyme
ACR: Albumin-creatinine ratio
BIPQ: Brief Illness Perception Questionnaire
CKD: Chronic kidney disease
CKD-EPI: Chronic kidney disease epidemiology collaboration
CRT: Cluster-randomised trial
DLR: German Space Agency
eCRF: Electronic case report form
eGFR: Estimated glomerular filtration rate
ESRD: End-stage renal disease
FU: Follow-up
G-BA: Federal Joint Committee
GP: General practitioner
HIS: Hospital information system
ICD-10: International Classification of Diseases, version 10
IMBI: Institute of Medical Biometry and Statistics, Medical Center-University of Freiburg
IMIBE: Institute for Medical Informatics, Biometry and Epidemiology; Department of Clinical Epidemiology; University Hospital Essen
IT: Information technology
KKSG: Knappschafts-Kliniken-Service-GmbH
KFRE: Kidney failure risk equation
NSAID: Non-steroidal anti-inflammatory drug
PI: Principal investigator
PIKS: Perceived Kidney Knowledge Survey
PRO: Patient-reported outcome
PZN: Central Pharmaceutical Number
RAAS: Renin-angiotensin-aldosterone system
SF12: Short Form 12
SGLT2: Sodium-glucose co-transporter 2
